# Critical review of the safety assessment of nano-structured silica additives in food

**DOI:** 10.1186/s12951-016-0189-6

**Published:** 2016-06-10

**Authors:** Hans Christian Winkler, Mark Suter, Hanspeter Naegeli

**Affiliations:** Institute of Pharmacology and Toxicology, University of Zurich-Vetsuisse, Winterthurerstrasse 260, 8057 Zurich, Switzerland; Immunology Division, Vetsuisse Faculty, University of Zurich, Winterthurerstrasse 260, 8057 Zurich, Switzerland

**Keywords:** Aerosil, Corona, Dendritic cells, E 551, Food toxicology, Lymphoid tissue, Nanomaterial, SAS, Synthetic amorphous silica, Silicium dioxide

## Abstract

The development of nano-materials is viewed as one of the most important technological advances of the 21st century and new applications of nano-sized particles in the production, processing, packaging or storage of food are expected to emerge soon. This trend of growing commercialization of engineered nano-particles as part of modern diet will substantially increase oral exposure. Contrary to the proven benefits of nano-materials, however, possible adverse health effects have generally received less attention. This problem is very well illustrated by nano-structured synthetic amorphous silica (SAS), which is a common food additive since several decades although the relevant risk assessment has never been satisfactorily completed. A no observed adverse effect level of 2500 mg SAS particles/kg body weight per day was derived from the only available long-term administration study in rodents. However, extrapolation to a safe daily intake for humans is problematic due to limitations of this chronic animal study and knowledge gaps as to possible local intestinal effects of SAS particles, primarily on the gut-associated lymphoid system. This uncertainty is aggravated by digestion experiments indicating that dietary SAS particles preserve their nano-sized structure when reaching the intestinal lumen. An important aspect is whether food-borne particles like SAS alter the function of dendritic cells that, embedded in the intestinal mucosa, act as first-line sentinels of foreign materials. We conclude that nano-particles do not represent a completely new threat and that most potential risks can be assessed following procedures established for conventional chemical hazards. However, specific properties of food-borne nano-particles should be further examined and, for that purpose, in vitro tests with decision-making cells of the immune system are needed to complement existing in vivo studies.

## Background

The use of nanotechnology has many potentially beneficial applications in food production, processing and storage. The largest share of predicted markets involves nano-sized coatings of food-packaging materials that optimize mechanical properties or exert antimicrobial activity. In the future, nano-sized additives may be deliberately included to modify food properties such as taste, sensation, color, texture, consistency or shelf life, to fortify basic foods with nutrients and vitamins or to enhance bioavailability. An emerging application in the food industry includes, for example, the use of nano-Fe particles for iron supplementation. Nano-sized materials might further be employed as indicators of food quality and freshness, or to ensure traceability [1–3]. In contrast to these novel developments, nano-structured silica has been on the market as a food additive since around 50 years. In the United States, the Food and Drug Administration allows up to 2 % by weight of such silica particles to be added to food [4]. Within the European Union (EU), Commission Regulation 1129/2011 sets a maximum level for silica of 1 % by weight in dried powdered foodstuffs [5]. Silica particles may thus be regarded as a paradigmatic case for the safety assessment of nano-material applications in the food industry.

A European Commission Recommendation defines nano-materials as having one dimension not exceeding 100 nm [6]. However, there is no solid scientific ground to propose a strict size boundary and the prefix “nano” does not make a substance automatically harmful. Nevertheless, the nano-size scale changes the material characteristics as compared to larger particles or the same substance in a dissolved state. Nano-sized materials display an increased surface-to-mass ratio that enhances their reactivity compared to larger structures [7, 8]. Also, nano-sized particles easily penetrate intact cell membranes thus conferring the potential for trafficking across biological barriers including the epithelium of the gastrointestinal tract [9–13]. Until now, the health effects of nano-particles have been studied mainly in relation to a respiratory uptake [14]. Considering their widespread food-related uses, however, there is an urgent need to review the suitability of oral toxicity and risk assessment studies addressing the long-term safety of nano-structured silica.

## Synthetic amorphous silica

Silicon (Si) is a metalloid displaying an atomic weight of 28. The terms “silicium” and “silica” refer to naturally occurring or anthropogenic materials composed of silicon dioxide (SiO_2_), which appears in two major forms, i.e., crystalline and amorphous. Synthetic amorphous silica (SAS) is widely applied to processed foods and registered by the EU as a food additive with the code E 551 [15]. The main purpose of SAS particles in the food industry is to prevent poor flow or “caking”, particularly in powdered products. SAS particles are additionally employed as a thickener in pastes or as a carrier of flavors, and also to clarify beverages and control foaming [16–18].

Silica particles exist in large amounts in nature and it is acknowledged that that they have been dietary constituents throughout human evolution. However, the risk assessment of silica discussed in this review is limited to man-made materials introduced as food additives. In 1942, Harry Kloepfer (a chemist working at Degussa, now Evonik) invented the Aerosil procedure for the production of SAS particles intended for the food industry [19, 20]. Following a standard pyrogenic process, also known as flame hydrolysis, silicon tetrachloride is burned in a hydrogen flame at temperatures of 1000–2500 °C, generating silica nano-particles with a diameter of ~10 nm [21]. This material is denoted pyrogenic or fumed silica referring to the above production method. In an alternative wet route of synthesis, nanostructured SAS particles denoted as precipitated silica, silica gel or hydrous silica, are produced from alkali metal silicates dissolved in water and reacted with sulphuric acid. In the EU, only synthetic particles obtained by these pyrogenic or wet processes are allowed as food additive [15]. All SAS products aggregate into larger particles with sizes in the order of 100 nm, which further agglomerate to form micron-sized structures [14, 22]. The term “aggregate” describes an assembly of particles held together by strong forces such as covalent or metallic bonds. “Agglomerates” of particles appear as a consequence of weak forces like van der Waals interactions, hydrogen bonding, electrostatic attractions or adhesion by surface tensions. SAS materials are hydrophilic but can be rendered hydrophobic, thus reducing their moisture uptake, by subsequent surface modifications.

## Oral toxicity studies using SAS particles

A synopsis of animal studies addressing the oral safety of SAS particles was published by the European Centre for Ecotoxicology and Toxicology of Chemicals (ECETOC) [23] and, more recently, by the Organization for Economic Co-operation and Development (OECD) [24]. No mortality or adverse signs resulted from acute exposure by single oral administrations of hydrophilic SAS particles to rodents at doses of up to 5000 mg per kg body weight. A sub-acute (28-day) study was carried out by oral gavage administration of hydrophilic SAS particles to Wistar rats. The daily doses ranged between 100 and 1000 mg/kg body weight. None of the monitored endpoints (clinical signs, food consumption, body weight, behavioral tests, hematology, clinical chemistry parameters, organ weights, macroscopic pathology and histological examinations) revealed any substance-related abnormalities [25].

In a sub-chronic (90-day) toxicity study carried out in Charles River rats with daily doses of up to 3500 mg/kg body weight, hydrophilic SAS particles included in the feed did not elicit systemic toxicity and did not affect growth rate, food consumption or survival [26]. Also, no macroscopic or microscopic changes were observed in post-mortem analyses of the organs of exposed animals. A more detailed 90-day toxicity study was conducted in Wister rats with in-diet administrations of hydrophilic SAS particles (up to 4000 mg/kg body weight daily). Endpoints included general condition and survival, behavior, water intake, food consumption, body weight, hematology, clinical chemistry, urinary analysis, organ weights, macroscopic pathology and histological examinations. As observed in the previous sub-chronic study, none of these parameters revealed any effects ascribed to SAS ingestion [27]. Sub-chronic dietary exposure studies were also carried out with hydrophobic SAS particles not permitted as food additive in the EU. In one case [28], no treatment-related abnormalities were reported except minimal changes in the thyroid gland morphology of male rats exposed to 2000 and 4000 mg/kg body weight daily. In another repeated dose toxicity study with hydrophobic SAS particles [29, 30], Wister rats were exposed via the diet for 5 weeks at 0 (control), 500 or 1000 mg/kg body weight per day, and for 8 weeks at progressively increasing SAS doses from 2000 to 16,000 mg/kg body weight per day. Animals in these high-dose group developed severe atrophy of the liver detected by microscopic examination, which was also observed to a milder degree in the 1000-mg/kg dose group.

Further effects on the liver were described in more recent reports, thus converging on a potential systemic hazard of SAS particles (Table 1). In one repeated oral toxicity study, BALB/c mice were exposed for 10 weeks to hydrophilic nano- or micron-sized silica particles prepared from rice husk (not permitted as food additive in the EU) [31]. The inclusion rate in feed was 1 % (wt/wt) translating to an expected oral uptake of 1500 mg/kg body weight per day. The animal group fed the nano-particles showed a significantly higher serum level of alanine aminotransferase (a biomarker of liver injury) compared to untreated controls or animals tested with micro-sized silica. In the histologic examination of tissues from mice exposed to nano-particles, but not in those exposed to micro-particles, there was an appearance of fatty liver characterized by abnormally frequent lipid droplets in hepatocytes. Further liver reactions were detected in a repeated dose toxicity study in Sprague–Dawley rats [32]. The animals were exposed via feed to hydrophilic SAS particles obtained from Evonik (denoted “Evonik-SAS”) or from the Joint Research Centre of the European Commission (denoted “JRC-SAS”). Both materials were produced by flame hydrolysis but differed in their surface area, i.e., 380 and 200 m^2^/g for “Evonik-SAS” and “JRC-SAS”, respectively. These particles were delivered with the feed at different daily doses (between 100 and 2500 mg/kg body weight) for 28 days, whereby the exposure was extended to 84 days for the highest dose groups. No treatment-related effects were observed after 28 days. However, following 84 days of exposure, the occurrence of periportal liver fibrosis was higher than in control animals (Table 1). This increase in the frequency of liver fibrosis was significant in the JRC-SAS-treated animals (p = 0.02) but slightly below statistical significance (p = 0.07) in the Evonik-SAS-treated group. The histological effects were not accompanied by changes in clinical chemistry. Notably, this experiment also included some immunological parameters like IgG and IgM levels in blood, lymphocyte proliferation, as well as cytokine release from in vitro activated lymphocytes. None of the tested immunological endpoints were affected in any of the dose groups.

**Table 1:** Oral repeated dose toxicity studies with amorphous silica yielding liver effects

Species	Study length	Effect	LOAEL	NOAEL	Reference
Wistar rats	5–8 weeks	Liver atrophy	1000 mg/kg body weight per day	500 mg/kg body weight per day	[29, 30]
Balb/c mice	10 weeks	Fatty liver	1500 mg/kg body weight per day	NA	[31]
Sprague–Dawley rats	12 weeks	Periportal liver fibrosis	810 mg/kg body weight per day	NA	[32]
Fischer rats	103 weeks	Reduced liver weight	1000 mg/kg body weight per day	500 mg/kg body weight per day	[33]

Delivery of particles was by inclusion into the feed. This table proposes *LOAEL* (lowest observed adverse effect level) and *NOAEL* (no observed adverse effect level) values that differ from those purported in previous risk assessment reports. Other oral repeated dose studies [25–27] did not elicit adverse effects

Long-term dietary studies in rats [33] were used for the risk assessment of human exposure. Groups of 40 Fischer rats were fed 0 (control), 1.25, 2.5 and 5 % (wt/wt) hydrophilic SAS particles for 103 consecutive weeks. The design of this chronic bioassay is outlined for the high-dose group in Table 2. The feed was not examined for possible nutritional imbalances [34]. There were no test substance-related effects on food consumption, overall survival, clinical laboratory or hematologic results and microscopic pathology findings. Liver weights were significantly reduced in the females fed 2.5 and 5 % SAS particles and this effect might be a consequence of the lower body weight attained in these two higher dose groups relative to controls and animals in the 1.25 % inclusion group. It is retrospectively not possible to distinguish whether the effect on liver weight represents an adverse reaction to SAS ingestion or whether it is an indirect consequence of a possible nutritional imbalance not directly related to SAS exposure. Notably, SAS-treated males displayed isolated cases of hyperplastic nodules in the liver and pheochromcytomas in the adrenal gland, but none of the control animals had such rare lesions. A long-term study in B6C3F1 mice involved groups of 40 animals fed 0 (control), 1.25, 2.5 and 5 % (wt/wt) SAS particles for 93 weeks [33]. Again, the feed was not examined for nutritional imbalances. The growth rate was significantly reduced in the mice of the high-dose group only at the end of the first 10 study weeks. Food consumption was significantly increased in the males fed 2.5 and 5 % SAS particles but no other substance-related differences came to light. The treatment had no effect on clinical chemistry, hematologic results and macroscopic as well as microscopic pathology findings. Recently, the European Commission Scientific Committee on Consumer Safety pointed out that these long-term studies in rodents cannot be considered as adequate for risk assessment because it is not clear whether the research was conducted under generally accepted guidelines and because the test material was not properly described [35].

**Table 2:** Outline of the chronic toxicity study with SAS particles carried out in rats

	Weeks of feeding						
	0	5	15	30	50	81	103
Body weight (g)	108 ± 6	174 ± 9	223 ± 11	253 ± 10	310 ± 18	364 ± 26	359 ± 56
Feed intake (g/day)		11.1 ± 0.4	10.4 ± 0.4	10.2 ± 0.5	11.9 ± 0.8	13.2 ± 0.8	12.7 ± 2.7
SAS intake (g/kg body weight per day)		3.2	2.3	2.0	1.9	1.8	1.8

Summarized data from the oral chronic toxicity study in Fischer rats [33]. This table illustrates the relationship between body weight, feed intake and daily doses in the females of the highest dose group, where the feed was supplemented with 5 % (wt/wt) SAS particles. When corrected for the actual feed intake, the daily dose of SAS particles was between 1.8 and 2.0 g/kg body weight during most of the study period

To summarize, a critical analysis of existing oral repeated dose studies in rodents reveals data gaps and uncertainties limiting their predictive value for the risk assessment of human dietary exposure. Some studies were based on poorly characterized particles in terms of composition, impurities or physico-chemical properties, and most reports lacked an assessment of particle size distribution.

## Oral bioavailability and systemic distribution

Little is known on the intestinal absorption of nano-sized SAS particles and the potential to disseminate into tissues. A limited systemic uptake following oral ingestion cannot be ruled out although none of the tested SAS particles were shown to bio-accumulate [19]. This is illustrated by a repeated dose kinetic study, in which rats were administered SAS particles via gavage at a low dose of 20 mg/kg body weight during 5 consecutive days [36, 37]. On the sixth day of the study, measurements by quadrupole inductively coupled plasma mass spectrometry (ICP-MS) revealed only slightly elevated silicon levels in liver and spleen. The gastrointestinal absorption after administration of SAS particles, estimated from these silicon measurements in tissues and not considering a concurrent excretion, ranged between 0.03 and 0.06 % of the total oral dose [38]. In another repeated dose study already presented above, rats were administered SAS particles via feed for 28 days, with continued administration in the high-dose group for up to 84 days. Silicon contents were measured at days 29 and 84 by ICP-MS in liver, spleen, kidney, brain and testis. Conversion of the resulting silicon levels to assumed silica concentrations in tissues, again not considering concomitant excretion processes, indicated an overall oral bioavailability of 0.02 % or less [32]. The highest silica concentration (up to ~300 mg/kg tissue against a physiologic background below the limit of detection) was found in the spleen of SAS-exposed animals. So far, no SAS particles have been detected in mesenteric lymph nodes or any other organ after oral uptake and, therefore, it is not clear whether the observed silicon residues exist in a particulate form or rather in a dissolved state, for example as orthosilicic acid.

Another key issue that has not yet been investigated with regard to bioavailability and systemic distribution is the effect of biomolecules bound to nano-particles changing their surface properties [38]. In particular, SAS nano-particles are known to be decorated by proteins, for example fibrinogen or apolipoprotein A1, as soon as they get in contact with biological fluids [39]. The term “protein corona” was introduced to describe the attachment of plasma proteins to the surface of nano-particles [40, 41]. One possible effect of this corona is that it mediates the uptake of nano-particles into cells and organs including the liver, thus influencing bioavailability and tissue distribution [42].

## Human exposure

To obtain realistic dietary exposure values for a European population, Dekkers and colleagues [18] selected food products from a local supermarket (ready-to-eat meals, soups, sauces, coffee creamers, pancake mixes, seasonings and supplements) based on declarations for the presence of E 551. Next, the total silica concentration in these products was determined by inductively coupled plasma atomic emission spectrometry (ICP-AES). Then, dietary intake estimates of these products for the population were calculated using a Dutch food consumption survey [43]. Based on estimated consumption and silica levels, the resulting daily dietary intake was 9.4 mg SAS particles per kg body weight. This total daily quantity includes all SAS particles regardless of their degree of aggregation or agglomeration. Hydrodynamic chromatography with inductively coupled plasma mass spectrometry (HDC-ICPMS) showed that up to ~40 % of SAS particles detected in food products display an external diameter <200 nm [18].

The next question was whether the SAS particles in commercial food products would be destroyed in the digestive tract or rather withstand gastrointestinal conditions. Using an in vitro system mimicking human gastric digestion, it could be demonstrated that nano-structured silica forms agglomerates under acidic conditions resembling the milieu of the stomach. By switching the conditions to those imitating the subsequent intestinal digestion stage, however, these large agglomerates disintegrate readily into nano-sized silica structures. In light of these in vitro findings, it was concluded that up to ~80 % of orally ingested SAS particles withstand gastric dissolution and display a nano-sized range once they reach the intestinal lumen [44].

## Inadequacies of existing risk assessments

Nano-particles are neither inherently toxic nor inherently safe and possible adverse effects should be tested case-by-case. In principle, a standard assessment with risk = hazard × exposure, which includes hazard identification, hazard characterization, exposure assessment and risk characterization, is applicable to nano-materials in food [21, 45, 46].

The United Kingdom Food Standards Agency Expert group on Vitamins and Minerals (EVM) performed an assessment of the oral safety of SAS particles, yielding a safe upper dietary level for daily consumption of 1500 mg SAS particles per day for adults [34]. This upper safety limit was calculated from the only available long-term toxicity study in rats [33], despite its limitations recently reiterated by the Scientific Committee on Consumer Safety [35]. The EVM experts noted that oral exposure to SAS particles for 103 consecutive weeks elicited effects on body weight and absolute liver weight (concerning the groups with a 2.5 and 5 % dietary inclusion rate). However, they attributed this outcome to possible nutritional imbalances, not adjusted in these two higher dose groups and considered unlikely to be relevant for humans. On this basis, the EVM group concluded that the no observed adverse effect level (NOAEL) is equivalent to the highest dose tested, i.e. 5 % SAS (wt/wt) in the diet. In the absence of further information or studies explaining the reduction in body and liver weight, we advocate a more cautious approach by deriving from the same study a lower NOAEL equivalent to the 1.25 % inclusion rate, also in view of the emergence of liver hyperplastic nodules and adrenal pheochromcytomas recorded in a few SAS-treated males but never in controls [33]. This more cautious interpretation is supported by a recent, above-described study [32] revealing periportal liver fibrosis in SAS-exposed rats.

As indicated, the EVM expert group opted for a NOAEL of 5 % dietary inclusion and further estimated that this translates by default to a daily dose of 2.5 g/kg body weight. After introducing an uncertainty factor of 100 (to adjust for inter-species as well as inter-individual variations in sensitivity), the derived safe upper level for lifetime daily consumption of SAS particles was 25 mg/kg per day, equivalent to 1500 mg per day for a 60-kg adult. In terms of elemental silicon, the “safe” upper limit for daily consumption is 12 mg/kg body weight per day (equivalent to 700 mg for a 60-kg adult). In 2004, the Scientific Panel on Dietetic Products, Nutrition and Allergies (NDA Panel) at the European Food Safety Authority concluded for silicon that “there are no suitable data for dose—response for establishment of an upper level” [47]. In 2009, however, the Scientific Panel on Food Additives and Nutrient Sources added to Food (ANS Panel) took into consideration the risk assessment carried out by the EVM experts and adopted their proposed upper limit of 1500 mg per day when assessing silicon dioxide as food additive [46]. It remains to be mentioned that default intake calculations made by translating a 5 % inclusion in the feed of rats to an assumed ingestion of 2.5 g/kg per day seem incorrect as the actual daily dose of SAS particles, determined from feed consumption in the high-dose group, was around 1.9 g/kg body weight during most of the study period (Table 2). Similarly, the lower inclusion rates of 1.25 and 2.5 % correspond to daily oral doses of around 0.5 and 1 g/kg, respectively. To summarize, several gaps in both study design and interpretation of results diminish the predictability of the only available long-term bioassays in rodents addressing the oral safety of SAS particles.

## Potential for local effects in the gastrointestinal tract

Depending on the nature of identified hazards that raise concerns, the canonical risk characterization may need to be complemented with endpoints that are not routinely assessed in the toxicological evaluation of chemicals. For example, a nano-Trojan horse hypothesis has previously been proposed in view of the observation that, in human lung epithelial cells exposed to Co_3_O_4_ or Mn_3_O_4_ nano-particles, the generation of reactive oxygen species (ROS) was higher than in controls exposed to an equivalent concentration of dissolved cobalt or manganese salts [10]. It is equally conceivable that the binding of luminal antigens to SAS particles could aid their delivery to reactive cells of the gastrointestinal tract. In this respect, we note in particular that none of the above-reviewed studies examined local effects on the lymphoid tissue of the gastrointestinal mucosa. Rather than causing ROS production, SAS particles have been implicated in the release of pro-inflammatory cytokines (see below).

Although the function of the immune system is to safeguard the host against invasive pathogens, the steady-state gastrointestinal tract is geared towards immune silencing or tolerance to avoid futile reactions to innocuous food antigens and beneficial commensal microorganisms [48, 49]. Upon oral exposure, foreign particles encounter a single layer of mucous membrane lining the digestive tract. This large vulnerable surface is defended by the gut-associated lymphoid tissue, which consists of loosely organized clusters of lymphoid cells and more organized Peyer’s patches. Nano-particles including those made of silica are known to penetrate this lymphoid tissue underlying the epithelial barrier [50–53], where they may disrupt the critical balance between tolerance to harmless food constituents and commensals on the one hand and inflammatory reactions towards pathogens on the other hand [54, 55]. Specific analyses of Peyer’s patches are not mentioned in the available sub-chronic and chronic toxicity studies in rodents and, therefore, it is not possible to ascertain whether delayed local effects on the gut-associated lymphoid system were adequately excluded.

Previous studies highlighted the fact that there is one critical site in which food-borne nano-particles accumulate during lifelong exposure, i.e., in “pigment cells” of the gut-associated lymphoid tissue where the earliest signs of inflammatory bowel disease (IBD) are noted (reviewed in [56–58]). This is a wide group of chronic conditions ranging from Crohn’s disease (affecting all segments of the digestive tract) to ulcerative colitis (restricted to the large bowel, [59]). The main gatekeepers of tolerance in the intestinal system, as well as major mediators of adverse reactions like IBD, are specialized antigen-presenting cells known as dendritic cells [60]. They act as scavengers of foreign materials by extending branched and rapidly changing projections across the epithelial barrier into the gut lumen and taking up particles by endocytosis [61]. Particles are also delivered directly to dendritic cells after their passage through microfold cells (M-cells) in the intestinal mucosa. In this way, dendritic cells filter out a volume of up to 1500 µm^3^, which equals their own cell volume, per hour [62]. Unlike other antigen-presenting cells, dendritic cells constitutively express class II major histocompatibility complexes and, in response to pathogen recognition, display co-stimulatory surface glycoproteins and produce inflammatory cytokines. For these reasons, dendritic cells constitute potent activators of the innate immune system and also polyvalent drivers of T lymphocytes [63, 64]. As nano-particles are in the same size range as many proteins or common viruses, it is not surprising to find that, by virtue of their function in probing the environment for intruding insults, dendritic cells capture nano-particles in an efficient manner [65–67].

It was shown that endotoxin-activated dendritic cells release the potent pro-inflammatory cytokine interleukin-1β (IL-1β) upon incubation with SAS nano-particles [68]. Mechanistically, this response has been linked to activation of the inflammasome complex, which in turn cleaves the pro-IL-1β precursor protein to release active IL-1β. In view of this finding, it will be of pivotal importance to determine how steady-state dendritic cells like those residing in the normal non-inflammatory intestinal mucosa react to the presence of food-borne SAS particles. IBD has a multi-factorial origin with genetic susceptibility, gut microflora and a dysfunction of the mucosal immune system as main drivers [60]. Additionally, various dietary factors have been implicated in the increasing incidence of IBD and several authors raised the concern that food-borne nano-particles may contribute to initiating this chronic inflammatory disease [69–71]. Inadvertent stimulation of the immune system by nano-particles could trigger a reaction sequence that abrogates tolerance to food constituents and commensal bacteria and thereby favor immune-mediated conditions with the hallmarks of IBD (reviewed by [72]).

## Conclusions

Previous and current controversies on hormone or antibiotic residues in food illustrate that nutrition is a highly emotional area in the public perception. The finding that SAS particles activate the inflammasome and, hence, are not biologically inert is intriguing because this type of nano-structured material has been employed since decades as food additive and is so far considered to be safe for consumers. Based on a critical review of existing oral toxicity studies, however, we consider that adverse effects from food-borne SAS particles cannot be excluded. Table 1 summarizes independent repeated dose studies that reached much the same conclusion with an oral NOAEL of 500 mg per kg body weight and a lowest observed adverse effect level (LOAEL) in the range of 810–1500 mg per kg body weight. Therefore, we advocate a prudent approach by taking the oral NOAEL of 500 mg/kg, based on body and liver weights in the 103-week feeding study in rats, as point of departure for the risk assessment of SAS particles. This NOAEL is lower than previously proposed [34] but, as summarized in Table 1, is consistent with the outcome (liver atrophy) of a 8-week feeding study and the liver effects (periportal fibrosis) in a 12-week feeding study, both in rats, at daily doses of 810–1000 mg/kg body weight. By applying a default uncertainty factor of 100 (to adjust for inter-species and inter-individual variations in sensitivity), this NOAEL would yield a safe upper level for the lifetime intake of SAS particles of 5 mg/kg body weight per day. The estimated consumption by a European population of 9.4 mg per kg body weight and day would, therefore, suggest that the dietary exposure to SAS particles should be reduced to remain, even with a worst case exposure scenario, below this newly proposed safety threshold of 5 mg/kg daily. Importantly, further studies are needed to investigate local effects of SAS particles in the gastrointestinal system, particularly on the gut-associated lymphoid tissue and embedded dendritic cells. In a broader perspective, mechanistic in vitro studies at different levels of biological complexity are necessary to understand in depth how food-borne nano-particles may influence the delicate balance between immune tolerance and inflammatory responses that depends on the proper function of dendritic cells in the intestinal mucosa. This research direction is crucial to eventually address the concern that the higher occurrence of IBD may be in part a consequence of the lifelong ingestion of nano-sized or nano-structured food additives increasingly used in the modern diet.

## Abbreviations

ANS: Scientific Panel on Food Additives and Nutrient Sources added to Food
E 551: synthetic amorphous silica (food additive)
ECETOC: European Centre for ecotoxicology and toxicology of chemicals
EU: European Union
EVM: Food Standards Agency Expert group on vitamins and minerals
HDC-ICPMS: hydrodynamic chromatography with inductively coupled plasma mass spectrometry
IBD: inflammatory bowel disease
ICP-AES: inductively coupled plasma atomic emission spectrometry
Ig: immunoglobulin
IL-1β: interleukin-1β
JRC: Joint Research Centre of the European Commission
LOAEL: lowest observed adverse effect level
NDA: Scientific Panel on dietetic products, nutrition and allergies
NOAEL: no observed adverse effect level
OECD: Organzation for Economic Co-operation and Development
ROS: reactive oxygen species
SAS: synthetic amorphous silica
